# 

**Published:** 1871-09

**Authors:** 


### Books and Pamphlets Received.

Practical Midwifery and Obstetrics, including Anæsthetics. By John Tanner, M.D·, M.A., L.L.D. Philadelphia: J. B. Lippincott & Co., 1871. Buffalo: Breed, Lent & Co.

A Manual of Midwifery, including the signs and symptoms of Pregnancy, Obstetric operations, diseases of the Puerperal state, &c., &c. By Alfred Meadows, M.D., London. First American from the second London edition, revised and enlarged with illustrations. Philadelphia: Lindsay & Blakiston, 1871. Buffalo: Breed, Lent & Co.

Handy-Book of the treatment of Women's and Children's diseases, according the Vienna Medical School, with Prescriptions. By Dr. Emil Dillnberger. Translated from the second German edition. By P. Nicol, M.D. Philadelphia Lindsay & Blakiston, 1871. Buffalo: Breed, Lent & Co.

Essay on growths in the Larynx: with reports, and an analysis of one hundred consecutive cases treated by the Author, and a tabular statement of all published cases treated by other practitioners since the invention of the Laryngoscope. By Morell Mackenzie, M.D., London, M.R.C.P., with numerous illustrations in Chromo-Lithography and Wood Engraving. Philadelphia: Lindsay & Blakiston, 1871. Buffalo: Breed, Lent & Co.

The Physicians Prescription Book, containing lists of terms, phrases, contractions and abbreviations used in Prescriptions, with explanatory notes, the Grammatical construction of Prescriptions, Rules for the pronunciation of Pharmaceutical terms, a Prosodiacal vocabulary of the names of Drugs, &c. By Jonathan Pereira, M.D., F.R.S. Fifteenth Edition. Philadelphia: Lindsay & Blakiston, 1871. Buffalo: Breed, Lent & Co.

Cancer: its classification and remedies. By J. W. Bright, M.D. Philadelphia: S. W. Butler, M.D., 1871.

Atlantic Monthly, Educational Monthly, Advance, N. Y. Observer, Newspaper Reporter, Phrenological Journal, Missionary Herald, Peterson's Musical Monthly.

Electrolysis, and its application to the treatment of disease. By A. D. Rockwell, A.M., M.D. New York: D. Appleton & Co.

Constitution and By-laws of the Monroe County Medical Society.

The Physical Diagnosis of Brain Disease. By Reuben A. Vance, M.D. New York: Wm. Baldwin & Co.

Report of the Resident Physician of Brigham Hall, a Hospital for the Insane, for the year 1870.

Containing Original Articles, Reports of Medical Societies and Hospitals, Editorial, Reviews, Correspondence, Army News, etc., etc.; including the usual variety of Medical Periodical Publications. Specimen copies sent on application.

Address, Buffalo Medical & Surgical Journal, Buffalo, N. Y.

TERMS OF SUBSCRIPTION, - - $3.00 PER YEAR, IN ADVANCE.

